# Dual Spinneret Electrospun Polyurethane/PVA-Gelatin Nanofibrous Scaffolds Containing Cinnamon Essential Oil and Nanoceria for Chronic Diabetic Wound Healing: Preparation, Physicochemical Characterization and In-Vitro Evaluation

**DOI:** 10.3390/molecules27072146

**Published:** 2022-03-26

**Authors:** Mohamed Ahmed Mohamady Hussein, Oguzhan Gunduz, Ali Sahin, Mariusz Grinholc, Ibrahim Mohamed El-Sherbiny, Mosaad Megahed

**Affiliations:** 1 Clinic of Dermatology, University Hospital of RWTH Aachen, 52074 Aachen, Germany; mmegahed@ukaachen.de; 2 Department of Pharmacology, Medical Research Division, National Research Center, Dokki, Cairo 12622, Egypt; 3 Center for Nanotechnology & Biomaterials Application and Research (NBUAM), Marmara University, Istanbul 34722, Turkey; oguzhan@marmara.edu.tr; 4 Department of Metallurgical and Materials Engineering, Faculty of Technology, Marmara University, Istanbul 34722, Turkey; 5 Department of Biochemistry, School of Medicine, Marmara University, Istanbul 34854, Turkey; alisahin@marmara.edu.tr; 6 Genetic and Metabolic Diseases Research and Investigation Center (GEMHAM), Marmara University, Istanbul 34854, Turkey; 7 Laboratory of Molecular Diagnostics, Department of Biotechnology, Intercollegiate Faculty of Biotechnology, University of Gdansk, 80307 Gdansk, Poland; mariusz.grinholc@biotech.ug.edu.pl; 8 Nanomedicine Laboratory, Center for Materials Science (CMS), Zewail City of Science and Technology, 6th of October, Giza 12578, Egypt

**Keywords:** nanoceria, dual spinneret electrospinning, PU/PVA-gelatin-based nanofibrous scaffolds, diabetic wound healing

## Abstract

In this study, a dual spinneret electrospinning technique was applied to fabricate a series of polyurethane (PU) and polyvinyl alcohol–gelatin (PVA/Gel) nanofibrous scaffolds. The study aims to enhance the properties of PU/PVA-Gel NFs loaded with a low dose of nanoceria through the incorporation of cinnamon essential oil (CEO). The as-prepared nCeO_2_ were embedded into the PVA/Gel nanofibrous layer, where the cinnamon essential oil (CEO) was incorporated into the PU nanofibrous layer. The morphology, thermal stability, mechanical properties, and chemical composition of the produced NF mats were investigated by STEM, DSC, and FTIR. The obtained results showed improvement in the mechanical, and thermal stability of the dual-fiber scaffolds by adding CEO along with nanoceria. The cytotoxicity evaluation revealed that the incorporation of CEO to PU/PVA-Gel loaded with a low dose of nanoceria could enhance the cell population compared to using pure PU/PVA-Gel NFs. Moreover, the presence of CEO could inhibit the growth rate of *S. aureus* more than *E. coli*. To our knowledge, this is the first time such nanofibrous membranes composed of PU and PVA-Gel have been produced. The first time was to load the nanofibrous membranes with both CEO and nCeO_2_. The obtained results indicate that the proposed PU/PVA-Gel NFs represent promising platforms with CEO and nCeO_2_ for effectively managing diabetic wounds.

## 1. Introduction

Diabetes mellitus is classified as a metabolic disease. It is estimated that the number of people with diabetes will increase to 552 million by 2030 [1]. Diabetes mellitus is accompanied by some serious complications, where diabetic ulcers are one of the major complications [2]. The current therapeutic approaches for the treatment of diabetic wounds are still non-significant [3]. Thus, there is a crucial need to fabricate and develop novel materials with an effective capability to heal diabetic wounds efficiently.

Wound dressings, such as foams [4], hydrogels [5,6,7], sponges [8], and nanofibers [9,10], have been widely demonstrated to investigate their efficacy and improve their properties to meet the needs for chronic and diabetic wound closure, including antibacterial activity, cell attachment and proliferation, collagen deposition, vascularization enhancing, and rapid closure time of wounds with no scar formation. A scaffold can act as an appropriate microenvironment for cell adhesion, proliferation, differentiation, and extracellular matrix (ECM) formation [8,9,10]. It is important to note that the microenvironment determined by the physical and chemical properties of a scaffold plays a crucial role in cell function and subsequent tissue regeneration. A variety of biomaterials are utilized to fabricate nanofibrous mats that mimic the structural geometry of ECM. Some unique characteristics could be possessed by nanofibers (NFs), including a high surface area to volume ratio and excellent porosity, which are essential for infiltration of nutrition, gas permeation, cellular attachment, and proliferation [11,12]. Other features are of crucial demand, such as mechanical integrity, surface adhesion, and biocompatibility. Combining different materials to produce the nanofibrous mat could introduce an ideal pavement that is likely to possess such important features.

Polyurethane (PU) has been reported for its biocompatibility, ion and gas permeability, tunability, and excellent self-healing properties. PU NFS was modified with micro- and nanoparticles CUO to produce durable and safe materials showing efficient antibacterial activity [13]. Therefore, PU-based scaffolds have been applied in various applications, including tissue engineering applications such as vascular tissue engineering, wound dressing, and biomedical implants [14,15,16]. However, PU has some shortcomings that limit its biomedical applications. Drawbacks, such as low biodegradability, surface adhesion, and poor mechanical integrity, make it unpleasant to use PU alone. A combination of different materials was demonstrated as an excellent approach to improve the properties of PU and introduce it as a promising pavement in tissue regeneration. Some studies have proposed incorporating metal oxides, such as zirconium dioxide [17] and copper oxide (CuO) [18]. The results showed enhanced cell attachment and proliferation in zirconium/PU and antibacterial activity for CuO/PU NFs. The mechanical properties were also improved. Propolis [19] and essential oils, such as lavender oil [20], were also introduced to be electrospun with PU. The plant extract was incorporated into a blended solution of carboxymethylcellulose/polyurethane (CMC/PU). The electrospun NFs showed significant improvement in cellular infiltration and proliferation, collagen deposition, neovascularization, and healing percentage rate in the extract/CMC/PU treated wounded animal group compared to the control treated group (CMC/PU) [21].

Gelatin (Gel) is a natural hydrophilic polymer derived from collagen, which is the most abundant protein in the body. Gel is known for its biocompatibility, availability, biodegradation, and non-immunogenicity. Such features are very important and are required for tissue regeneration and engineering. The limitations and restrictions of Gel applicability are attributed to its fast degradation and poor mechanical property [22]. Polyvinyl alcohol (PVA) is a well-known non-toxic biocompatible and affordable synthetic polymer. PVA has been blended with various natural polymers, such as chitosan, gelatin, collagen, or alginates, to obtain new products showing good biocompatibility, with appropriate mechanical properties [23,24,25,26,27].

Nanoceria (nCeO_2_) were demonstrated in previous studies for tissue regeneration. As a result of their excellent biological properties, antioxidant, anti-inflammatory, and angiogenic proprieties, the nanoceria (nCeO_2_) were loaded into various polymeric scaffolds for tissue engineering and regeneration [28]. nCeO_2_ incorporated into alginate–gelatin scaffolds were applied to bone regenerations, where they enhanced osteoblast differentiation by scavenging free radicals [29].

Cinnamon essential oil (CEO) can be obtained from the bark of a plant. It displayed a broad-spectrum antimicrobial effect against molds, yeast, and bacteria through its major active constituent, cinnamaldehyde [30]. Natural CEO is used in a wide variety of fields in spices, food medicine, cosmetic products, and agricultural chemicals due to the presence of chemical constituents, including cinnamaldehyde (70–88%), *o*-methoxycinnamaldehyde (4–12%), cinnamyl acetate (CAc, 0.5–10%), and benzaldehyde (0.5–1%) [31,32]. Two challenges stand against bringing CEO, or essential oils (EOs) in general, to biological applications or clinical trials. The lower solubility of CEO in aqueous preparation hinders the efficient delivery of its active components to the target. The second challenge is that CEO is volatile in nature, which causes a significant loss during application [33,34]. Therefore, the incorporation of CEO into the electrospun NFs would help to avoid such drawbacks.

The trends toward electrospun blends, composite, or coaxial of synthetic/synthetic or synthetic/natural polymers are increasing. Previous studies have produced a wide variety of electrospun polymeric blends or multilayer-based NFs, such as PCL/Gel [22], PLGA/Gel [35], chitosan/PVA [36], polyethylene oxide/chitosan [37], PVA/PVP [38], PU/Gel [39], and PU/PVA [40]. Producing essential oil-loaded PU-based NFs was performed in a previous study [20]; however, we think that using PU as a monolithic NF could not be convenient because of their poor biodegradability. Therefore, we suggest producing PU NFs electrospun simultaneously with biodegradable polymeric NFs composed of PVA/Gel. Such multilithic-based NFs would ensure the improvement in the swelling, degradability, and applicability of the produced PU/PVA-Gel based NFs than using only PU-based NFs as a drug carrier system. At the same time, electrospinning PVA/Gel along with PU would avoid or limit the fast degradability of both PVA and Gel.

This study aims to design and develop a dual-jet electrospinning protocol that enables the production of dual spinneret electrospun NFs of PU and PVA/Gel containing bioactive agents for tissue regeneration in diabetic wounds. A dual spinneret electrospinning technique was applied to fabricate the proposed nanofibrous scaffolds. The study plan uses a series of composite nanofibrous scaffolds composed of PU/PVA-Gel incorporated with CEO to potentiate the cell proliferation of NFs loaded with a low dose of nanoceria (nCeO_2_). Nanoceria at 1–2% was reported for its efficiency as a cell proliferative agent used for tissue regeneration [41,42,43]. Below this concentration, the efficiency of nCeO_2_ as a proliferative agent is reduced. As we tried to explore the impact of nanoceria incorporated into polymer-based NFs; PLA/PVA/PLA trilayer NFs were designated, and they showed better impact when loaded with 1–2% nanoceria rather than 0.5% [39]. In this study, we try to enhance the physicochemical and biological properties of such a low dose of nanoceria by incorporating it along with CEO into new electrospun polymer-based NFs. The PU NF layer is electrospun simultaneously with the nanofibrous layer of polyvinyl alcohol/gelatin (PVA-Gel) via a dual spinneret electrospinning technique. Both nCeO_2_ and CEO are embedded in the PVA-Gel and PU layers, respectively. This design would provide convenient platforms for interactive wound dressing mats displaying both antibacterial and cell proliferative activity. Also, electrospinning of PVA-Gel and PU as one nanofibrous membrane will produce a wound dressing mat with good swelling, biodegradability, and mechanical properties. The embedding of EOs in PU would likely improve its electrospinnability. To our knowledge, this is the first time such a nanofibrous membrane composed of an NF layer (PU) along with an NF layer (PVA-Gel) has been produced. The first time was to incorporate both Ce NPs and CEO in nanofibrous scaffolds. This study plans to develop multifunctional electrospun fiber membranes and investigate their mechanical properties, chemical composition by FTIR, thermal stability, and morphology by SEM. It will also study the antibacterial activity and cytobiocompatibility of the produced NFs.

## 2. Materials and Methods

### 2.1. Materials

Cerium nitrate hexahydrate (Ce(NO_3_)_3_·6H_2_O) obtained from Sigma-Aldrich, with a purity exceeding 99%, was used as the precursor material. Cinnamon essential oil (CEO) was obtained from a local store. Gelatin (Gel, from bovine skin, Type B, Gel strength ~225 g bloom), polyurethane (PU), polyvinyl alcohol (PVA) with molecular weight (Mw = 85,000–124,000), and 87–89% hydrolyzed was purchased from Sigma-Aldrich. Tetrahydrofuran (THF) and dimethylformamide (DMF) were purchased from Fisher Scientific, Atlanta, GA. All other chemicals were of analytical grade and were used without further purification. MilliQ water (18.2 Ω  cm^−1^) was used to prepare all aqueous solutions.

### 2.2. Preparation of Cerium Oxide Nanoparticles (nCeO*_2_*)

Nanoceria was synthesized as reported in a previous study using cerium nitrate (Ce(NO_3_)_3_·6H_2_O) as a precursor [44]. A solution of cerium nitrate was prepared and stirred for a while, and then a determined amount of aqueous ammonia was added to obtain a light yellow precipitate. The obtained suspension was then centrifuged and washed three times and then dried in an oven at 60 °C overnight.

### 2.3. Physicochemical Characterization of Nanoceria

UV-Vis spectroscopy was used to record the electronic absorption spectra of the developed nCeO2. Scanning electron microscope (SEM, EVO LS 10, ZEISS, Jena, Germany) imaging was used to observe the shape and distribution of the nanoceria. SEM energy-dispersive X-ray spectroscopy (SEM-EDX) was employed to confirm the biosynthesis of nCeO_2_. The morphology and size of nCeO_2_ were recorded by transmission electron microscope (JEOL TEM-1011) imaging also was performed at an accelerating voltage of 100 kV. The TEM sample was prepared by depositing 10 μL of the colloidal suspensions on a 200 mesh formvar/carbon film-coated Cu grid, followed by air drying. The excess film was removed with absorbent paper. Histograms of the NP size distribution were generated with ImageJ^®^. Fourier transform infrared (FTIR) spectra were recorded using a 4700 FTIR spectrometer. The data were normalized to the background spectrum recorded from a clean, empty cell. The dried powder of Ce NPs was placed in the sample holder of the spectrometer, where the IR-spectra were taken in the range of 4000–400 cm^−1^ with a resolution of 4 cm^−1^, at 25 °C.

### 2.4. Preparation of Electrospinning Solutions

The PU-CEO/PVA-Gel-nCeO_2_ dual-fiber membranes were fabricated by the electrospinning technique. A 12% (*w*/*v*) PVA solution was prepared in distilled water (DW) with heating at 90 °C and stirring for 4 h. The polymer solution was then left for cooling. Gel solution 10% was prepared in DW at 60 °C for 1 h. Gel was then added to PVA solution (with a Gel/PVA ratio of 3/7) and stirred for 2 h at 50 °C until a homogenous solution was obtained. Then 0.5% of nCeO_2_ was added to the polymer solution and left under stirring for 2 h to obtain a homogenous PVA-Gel-nCeO_2_ solution. PU solution (12%) was prepared by dissolving in dimethylformamide (DMF), and tetrahydrofuran (THF) in a 1:1 volume ratio, which contained CEO, and then left for stirring overnight.

### 2.5. Fabrication of PU-EO/PVA-Gel- nCeO*_2_* Electrospun Membranes

**Scheme 1:**
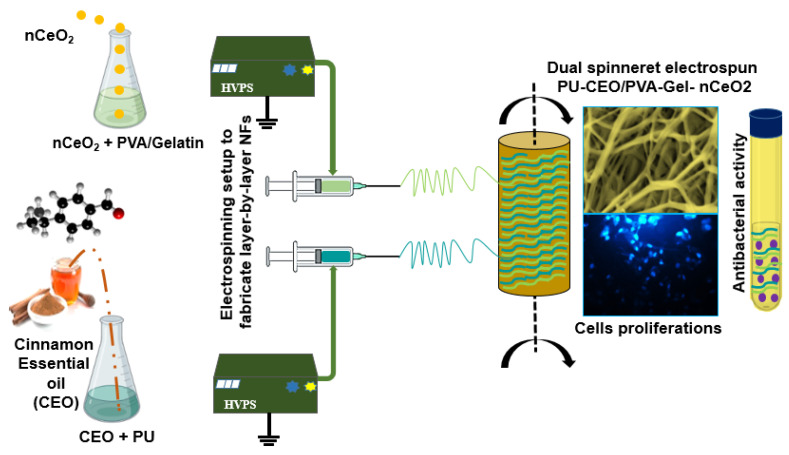
Schematic representation of the setup for the dual spinneret electrospun scaffolds PU-CEO/PVA-Gel-nCeO_2_.

The PU/PVA-Gel dual-fibers incorporated with CEO and nCeO_2_ were fabricated, as shown in Scheme 1. Both PU-CEO (10 mL) PVA-Gel-nCeO_2_ (10 mL) were electrospun at a tip to collector distance of 13 cm. The applied flow rate and voltage were 0.2 mL/h and 25 kV, respectively. Fibrous membranes deposited on the collector were carefully removed and stored in a desiccator at room temperature until used. Bare (nCeO_2_/CEO free) electrospun PU/PVA-Gel NFs membranes were named pure-S1. Membranes with 0.5% (*w*/*w*) loaded nCeO_2_ were assigned as nCeO_2_-S1, where the membranes loaded with both CEO and nCeO_2_ was named CEO/nCeO_2_-S3 and CEO/nCeO_2_-S4, as shown in Table 1.

### 2.6. Mechanical, Chemical, and Morphological Properties

To study the mechanical properties of the pure-S1, nCeO_2_-S2 and CEO/ nCeO_2_-S3, the samples were cut into dimensions of 1 cm × 5 cm. The thickness of the electrospun NFs was measured using a digital micrometer (Mitutoyo MTI Corp., New York, NY, USA). Mechanical tests were performed by recording tensile strength and elongation at break using a tensile test machine (SHIMADZU 5 KN, Kyoto, Japan). The upper and lower portions of each sample were located horizontally in the relevant section of the device. Both ends of each specimen were compressed by the top and bottom grips and subjected to a tensile test under conditions of 5 mm/min test speed. The results were analyzed using Bluehill 2 software (Elancourt, France). An FTIR 4700 model machine (JASCO 4700, Jasco Manufacturing, Portland, OR, USA) was used to record the chemical composition of the prepared hybrid NFs. It was performed at room temperature over the range of 4000–400 cm^−1^ in the transmission mode with a resolution of 4 cm^−1^ (32 scans). The morphology of the fibrous membranes was observed by high-resolution SEM (FEI QUANTA FEG SEM 450), which is a state-of-field emission SEM (FE-SEM). The imaging process was carried out using a large field detector (LFD). Nonconductive samples were coated with gold, and imaging was performed at 10 kV voltage, 4–10 mm distance, and 100 Pa pressure. The images were analyzed with image analysis software (ImageJ, National Institutes of Health, Stapleton, NY, USA) to determine the average fiber diameter. The porosity assessment of the prepared NFs was performed by the liquid displacement method using Equation (1), as previously reported [45,46,47]. Briefly, the mats with specific amounts were immersed in a graduated cylinder containing the initial volume of ethanol (*V*1). Then, the resulting volume was measured (*V*2), and finally, the scaffold was removed from the ethanol and the residual ethanol volume was measured (*V*3). The volume changes before and after soaking were related to the porosity of the NFs.

(1) $$\mathrm{Porosity}\;\%\;=\frac{V1-V3}{V2-V3}\times 100$$

where *V*1 is the initial volume of ethanol, *V*2 is its volume after NFs soaking (where ethanol filled the pores), and *V*3 is the volume of the ethanol after the scaffold removal.

The thermal behavior of the NFs was recorded by differential scanning calorimetry (DSC, Shimadzu-Japan). A 10–12 mg sample was sealed in an aluminum pan and heated in the range between room temperature and 300 °C at a heating rate of 10 °C per minute in the presence of nitrogen as an inert carrier gas.

### 2.7. Biodegradability and Swelling

The swelling profiles of pure-S1, nCeO_2_-S2 and CEO/ nCeO_2_-S3 NF mats were investigated. NFs were weighed (*Ms*), placed in PBS (pH 7.4), and left in a shaking incubator at room temperature. The samples were removed from the buffer solution at specific time intervals, plotted with dry filter paper to remove any excess PBS, and weighed again (*Md*) until no change in weight was observed. The swelling percentage was calculated using Equation (2).

(2) $$\mathrm{Swelling}\;\%\;=\left[\frac{Md-Ms}{Md}\right]\times 100$$

The biodegradability of the produced NF mats was studied by placing pre-weighed NF samples in PBS (pH 7.4) in a shaking incubator for 14 days. The media were changed and replaced with fresh media every day. The NF samples were collected from the PBS medium, washed with distilled water, and dried at 50 °C in an oven until a constant weight was obtained. The remaining weight percentage was monitored daily using Equation (3), where *Wi* is the initial weight, and *Wf* is the final weight measured each day.

(3) $$\mathrm{Biodegradability}\;\%\;=\left[\frac{Wi}{Wf}\right]\times 100$$

### 2.8. Antibacterial Activity

The antibacterial efficacy of the membranes against *Staphylococcus aureus* (*S. aureus*, Gram-positive bacterium) and *Escherichia coli* (*E. coli*, Gram-negative bacterium, was evaluated quantitatively, as stated in ASTM 2149-01 standard. Nanofibrous mats were prepared and radiated under UV light for 30 min. The bacterial density was standardized using the McFarland scale to reach approx. 5 × 10^6^–10^7^ CFU/mL. For the antimicrobial evaluation, the specimens were placed in an inoculated buffer solution inside sterilized tubes, including bacterial suspension, followed by incubation overnight in a shaking incubator 150 rpm at 37 °C. Thereafter, one sample was removed from each tube, serially diluted, inoculated onto an agar plate, and incubated overnight at 37 °C. The CFU on agar plates were counted. Then, the percentage of survived bacteria associated with the sample was calculated based on Equation (4).

(4) $$\mathrm{Bacterial}\;\mathrm{survival}\;\%\;=\frac{B}{A}\times 100$$

where *A* and *B* are the numbers colony forming units (CFU) in the control and test samples, respectively. The experiments were replicated three times for each sample.

### 2.9. Cytotoxic Study

#### 2.9.1. MTT Cytotoxicity Assay

Adipose-derived mesenchymal stem cells (ADMSC, ATCC-PCS-500-011) at 5 × 10^3^ were seeded onto the scaffolds in the 96-well plates, as in the standard cell seeding procedure, after 4 h ultraviolet (UV) light sterilization. At the same time, monolayer cell cultures were incubated with the same number of cells in 150 μL as a control. The cell-scaffold constructs and monolayer cultures were incubated at 37 °C, 5% CO_2_ for 1 and 3 days in a humidified incubator (NuAire). The toxic effect of scaffolds was checked on days 1 and 3. To investigate cytotoxicity at a given time point, the MTT (3-(4, 5-dimethylthiazolyl-2)-2, 5-diphenyltetrazolium bromide) (Glentham Life Sciences) cytotoxicity assay method was used according to the manufacturer’s protocol. The absorbance value of the cytotoxicity test was measured at 570 nm wavelength (690 nm as Ref. value) in an ELISA reader (Enspire, Perkin Elmer, Waltham, MA, USA). The assay was studied 3 times, and the average of the results was considered the final result.

#### 2.9.2. Fluorescence Imaging

The samples were placed in the wells of 6-well cell culture plates and sterilized for 4 h by UV irradiation. ADMSCs were seeded in plastic dishes and incubated for 3 days in a humidified incubator at 37 °C with 95% air and 5% CO_2_. By the end of day 3, the medium of the cells was removed, and washed with 100 μL pre-warmed PBS three times. Following this, the cells were initially fixed with 4% formaldehyde (Sigma) at room temperature for 15 min, washed, and incubated with 0.1% Triton X-100 (Merck) to increase permeability for 10 min. Further, 1/5000 4′,6-diamidino-2-phenylindole (DAPI, Invitrogen) was added to each sample and kept for 5 min at room temperature to stain the nucleus of the cells. At the end of this step, samples were placed on the slide, and 2–3 drops of fluoromount were added. The results were observed and captured under a Florescence inverted microscope (Leica).

### 2.10. Statistical Analysis

The results were analyzed using GraphPad Prism 7 software (version 8.02). The statistical significance was evaluated by one-way ANOVA, (* *p* < 0.05, ** *p* < 0.01, *** *p* < 0.001). The results are expressed as means ± SD.

## 3. Results and Discussion

### 3.1. Characterization of nCeO*_2_*

**Figure 1:**
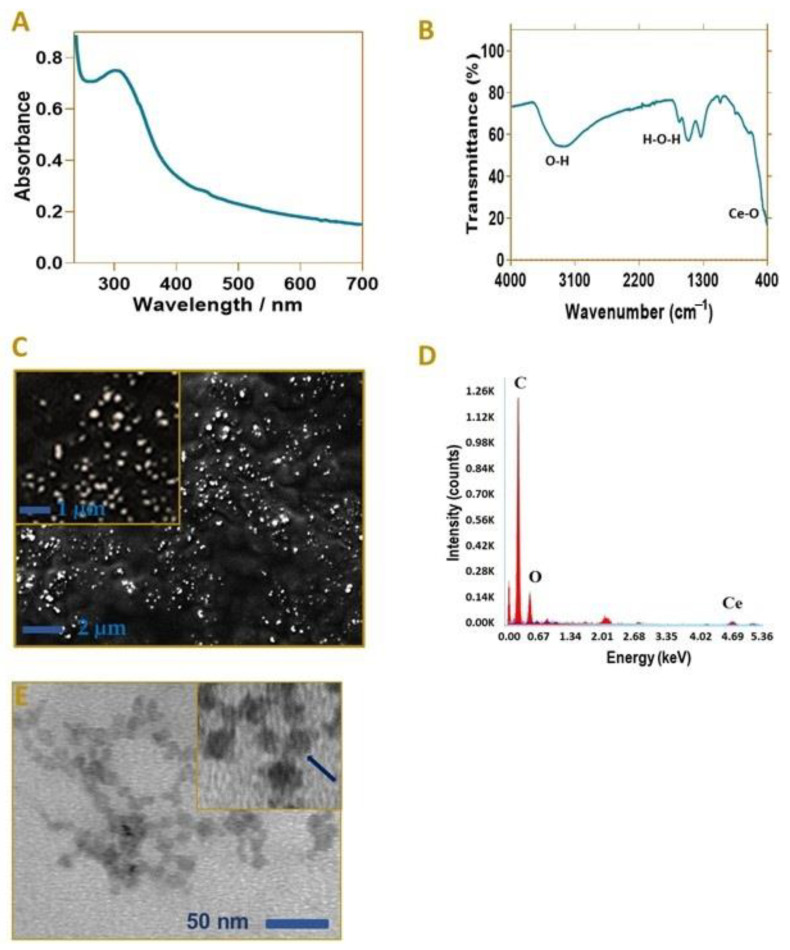
Nanoceria characterization by (**A**) UV-spectra of nanoceria, (**B**) FT-IR (4000–400 cm^−1^), (**C**) SEM, (**D**) EDX, and (**E**) TEM micrograph displays spherical-ovoid shaped nCeO_2_.

As shown in Figure 1A, the UV spectra of nanoceria were monitored where the absorbance peak was observed at 303 nm. Previous studies demonstrated the absorbance spectra of nCeO_2_ in the range of 290–360 nm [48,49]. In another study, the UV-spectra of nanoceria was conducted and detected in the range of 304–320 nm [50]. The SEM micrograph (Figure 1D) of nCeO_2_ displayed that particles are well distributed, and they were uniformly synthesized. TEM imaging was also performed, as shown in Figure 1E, to detect the shape and size of nCeO_2,_ where they appeared as spherical-ovoid-shaped particles in the range of 12–18 nm. The IR spectra were investigated in the range of 4000–400 cm^−1^, as noticed in Figure 1B, to confirm the synthesized nCeO_2_. The main characteristic peak attributed to the stretching band of Ce with O (Ce-O) can be observed at 441 and 622 cm^−1^. The peak, which is broad and located at 3253 cm^−1^ is assigned to the vibration of –OH. The band attributed to the flexion of H-O-H is observed to overlap with the band associated with O-C-O stretching, where they can be recorded at 1629 cm^−1^. Due to the bending vibration of H_2_O, there are bands that appeared at 1332 and 1060 cm^−1^ [48,51,52]. To confirm the synthesized nanoceria (nCeO_2_), EDX spectrum analysis was also employed, as shown in Figure 1C. The presence of Ce and O confirmed that the pure cerium oxide NPs were successfully synthesized.

### 3.2. SEM of CEO/nCeO*_2_* Loaded PU/PVA-Gel NFs

**Figure 2:**
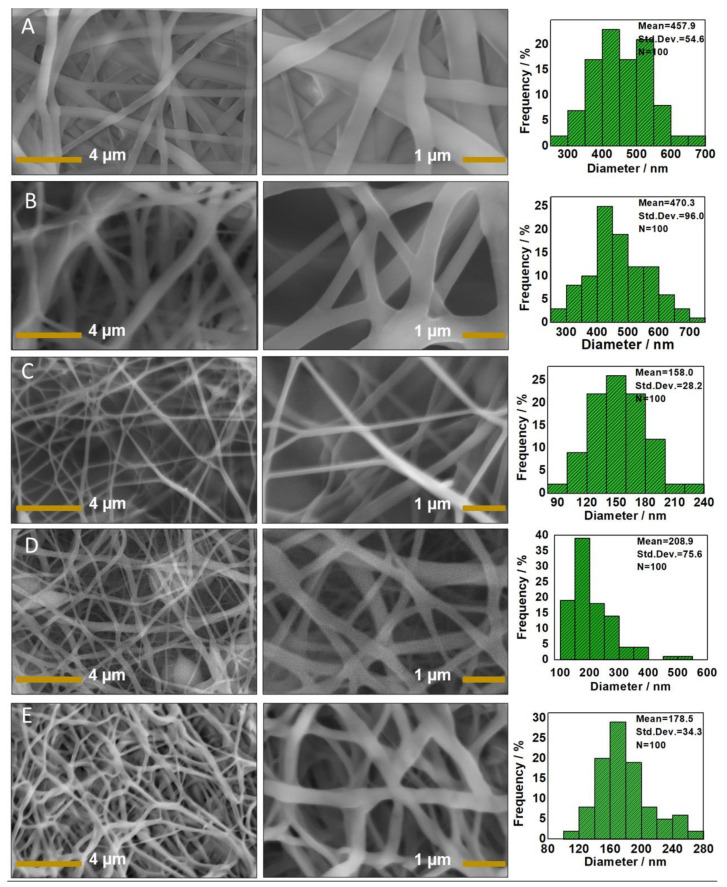
SEM micrographs of the developed NFs: (**A**) PU NFs; (**B**) Neat layer-by layer PU/PVA-Gel NFs (Pure-S1); (**C**) nCeO_2_ NP-loaded PU/PVA-Gel NFs (nCeO_2_-S2); (**D**) CEO/nCeO_2_-loaded PU/PVA-Gel NFs (CEO/ nCeO_2_-S3); and (**E**) CEO/nCeO_2_-loaded PU/PVA-Gel NFs (CEO/nCeO_2_-S4).

Figure 2 shows the SEM pictures and fiber diameter histograms of the nanofibers loaded with nanoceria and CEO. The monolithic PU NFs were imaged to show the differences. The PU NFs showed uniform fibers with an average diameter of 457.9 nm. The average diameter of the pristine PU/PVA-Gel fibers was 470.3 ± 96.0 nm. By adding nCeO_2_ (S2), the fiber diameter decreased to 158.0 ± 28.2 nm. The incorporation of a 1% CEO in S3 caused an increase in the fiber diameter to 208.9 ± 75.6 nm. By increasing the CEO to 3% (S4), the average diameter was 178.5 ± 34.3 nm. The embedding of NPs into the polymer solution is likely to increase the conductivity and its electrospinability with a decrease in the diameter of the fibers [53,54,55]. An increase in CEO concentration might enhance the solution’s viscosity and subsequently increase the fiber diameter [20].

The porosity of the scaffolds is beneficial for wound healing as it maintains homeostasis at the wound area, promotes moisture, ensures enough gas and nutrient exchange, and prevents pathogen penetration [56,57]. As shown, the porosity of the NFs was recorded in the range 73.5–79.6%, where the porosity decreased by incorporation of nCeO_2_ and CEO. This was reported in previous studies, which observed that the incorporated bioactive agents into the polymer-based NFs decreased their porosity compared to the non-loaded NFs [58]. According to previous studies, engineered membranes with 60–90% porosity could be suitable for tissue regeneration where such a porosity range is ideal to facilitate cell penetration and proliferation at their structure [2]. Ag NPs/chitosan dressing with 63–68% porosity was applied as a potential dressing for burns and chronic and diabetic wounds [59]. PCL/gelatin elcetrospun NFs with 60–70% porosity were applied for wound healing, where they showed good porosity for cellular penetration and in-growth [60]. In our study, porosity was appropriate for skin tissue engineering applications and wound healing. Furthermore, the gradual dissolution of the co-electrospun PVA/Gel layer and the deformation and elongation of Gel during application will leave NFs with micro-pores, which give more space opening for cellular penetration and migration [56,61]. Thus, the porosity of the drug-loaded NFs was optimum to perform their function for nutrient and gaseous exchange and to prevent the penetration of the external pathogens.

### 3.3. Swelling and Biodegradability

**Figure 3:**
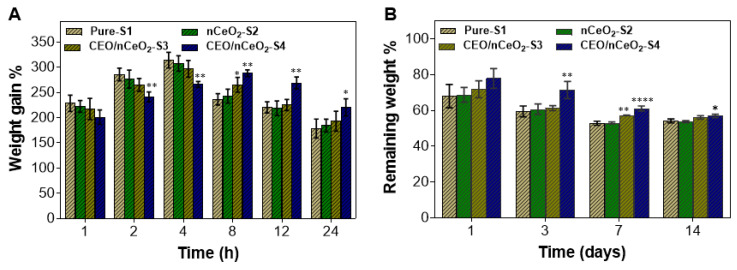
Water absorption, swellability (**A**), and biodegradability properties (**B**) of the developed dual spinneret electrospun Pu/PVA-Gel mats. Data are expressed as average ± SD. * *p* < 0.0332; ** *p* < 0.0021, **** *p* < 0.0001, versus control.

Demonstrating the swelling properties of the studied NFs is important to evaluate their capacity to absorb the exudates of the wounded skin. Figure 3A shows the swelling power of pure-S1, nCeO_2_-S2, CEO/nCeO_2_-S3, and CEO/nCeO_2_-S4. As it is displayed, there are 3 stages of the swelling pattern: rapid swelling, then slow swelling and ending by the plateau stage. It can be observed that both pure-S1 and nCeO_2_-S showed the highest swelling capacity within the first 4 h, where the swelling% increased to 314% and 307% of the initial dry weight, respectively. Meanwhile, the swelling capacity of CEO/nCeO_2_-S3 was similar, with a slight decrease in its swelling capability. On the other hand, the CEO in CEO/nCeO_2_-S4 was likely to cause less swelling due to the hydrophobic nature of the essential oil. However, after 4h, the increase in the swelling% continues gradually in CEO/nCeO_2_-S4. Meanwhile, the swelling% dropped in both pure S2 and nCeO_2_-S2. The decrease in the swelling capacity of pure-S1 and nCeO_2_-S2 is attributed to the partial loss of the PVA-Gel layer. Removing the excess exudates via the produced NFs is necessary to avoid septic shock caused by microbial ingrowth. The PVA/Gel polymeric NF layers, known for their high water uptake, can ensure the presence of such a moist environment, which is required for reepithelialization and local tissue remodeling.

A biodegradability profile was conducted to demonstrate the degradability and decomposition rates of the developed NFs. Figure 3B shows the degradation rates of the developed pure-S1, nCeO_2_-S2, CEO/nCeO_2_-S3, and CEO/nCeO_2_-S4 in PBS (pH 7.4). It is noted that CEO/nCeO_2_-S4 displayed the lowest degradation rate throughout the study. After 1 day, the remaining weight of CEO/nCeO_2_-S4 was 77.9%, while it was 68, 68.8, and 71.8% for pure-S1, nCeO_2_-S2, and CEO/nCeO_2_-S3, respectively. After 3 days, the degradation rate of CEO/nCeO_2_-S4 was still low; meanwhile, it increased in the period from 3 to 7 days with a remaining weight of 61.1%. The lower degradation rate of CEO/nCeO_2_-S4 after 1 and 3 days could be attributed to the tightly attached layers of PU-CEO and PVA/Gel-nCeO_2_. However, by the time and after a few days, this tight attachment is lost gradually as a result of the release of the CEO, which then causes a higher degradation rate for the NF mats of CEO/nCeO_2_-S4. Thus, after 14 days, the remaining weight of CEO/nCeO_2_-S4 was slightly higher than other samples. It was observed that weight loss increased slightly or decreased slowly after a week of the study. This can be attributed to the absorption of ions from the PBS medium, as observed in previous studies. 

### 3.4. Mechanical Properties

**Figure 4:**
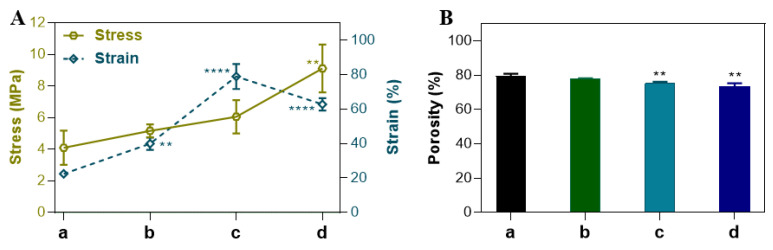
(**A**) Mechanical properties and (**B**) porosity% of (a) Pure-S1; (b) nCeO_2_-S1; (c) CEO/nCeO_2_-S3; (d) and CEO/nCeO_2_-S4. Data are expressed as average ± SD. ** *p* < 0.0021, **** *p* < 0.0001, versus control.

Performing the mechanical patterns of nanofibers is highly demanded for their feasibility in biomedical applications [10]. The mechanical properties of the nanofibrous membranes were evaluated by measuring their tensile strengths. Figure 4A shows the tensile capacities of the produced NFs through elongation at break. The tensile strength of pure-S1 mats was 4.1 ± 1.08 MPa, with elongation at break 22.4 ± 1.31%. The incorporation of nanoceria (nCeO_2_-S2) increased its strength to 5.16 ± 0.41 MPa, and the elongation to 39.9 ± 3.63%. The CEO/ nCeO_2_-S3 mats displayed strength at 6.05 ± 1.05 MPa with elongation at break at 78.85 ± 7.23%. The elongation at break of CEO/nCeO_2_-S4 was 62.71 ± 3.6%, with a strength of 9.12 ± 1.52 MPa. The results revealed that the tensile strength of Ce NPs and CEO-containing mats (S3 and S4) displayed a maximum tensile strength compared to others. It was reported that fibrous mats with a tensile strength in the range of 0.8 to 18.0 MPa can be optimum for biological application and wound healing [62].

### 3.5. FTIR Study

**Figure 5:**
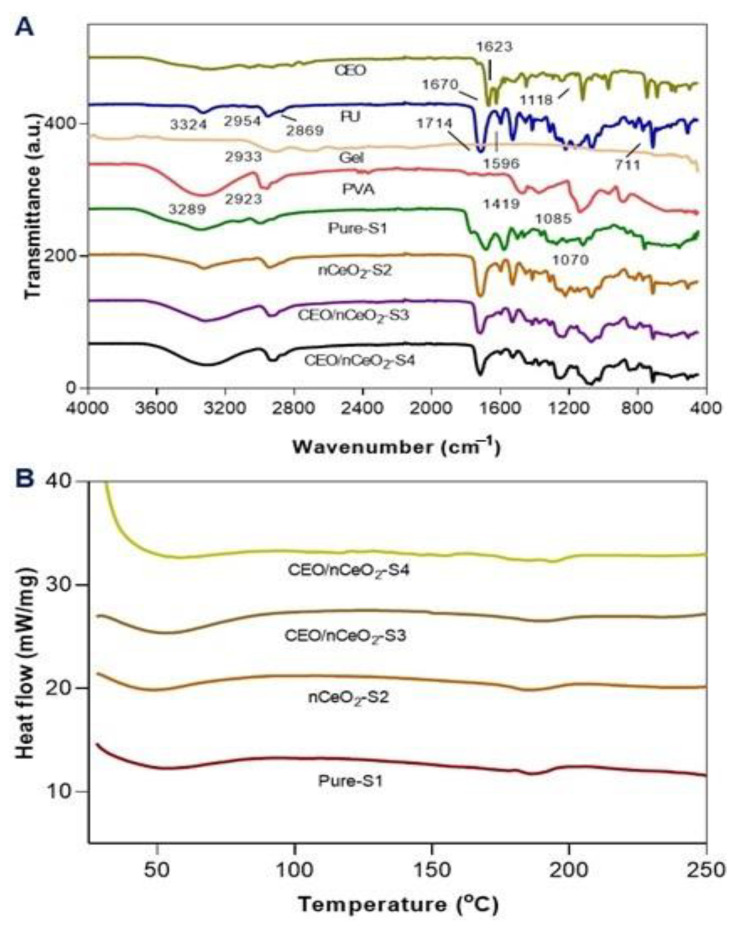
(**A**) FT-IR (4000–400 cm^−1^), and (**B**) DSC of the produced nanofibrous mats.

Chemical composition using FTIR was performed to confirm the fabrication of PU/PVA-Gel hybrid NFs. Figure 5A shows the FTIR of the NFs produced. For the spectroscopy of CEO, the characteristic bands at 1670 and 1623 cm^−1^, can be attributed to the carbonyl group (C=O) and unsaturated vibration peak of the benzene ring, respectively. The peak at 1118 cm^−1^, is assigned to the stretching of C–O–H of the phenolic compounds [63].

The spectra of nCeO_2_ showed a large absorption band at 450–625 cm^−1^, assigned to the vibrational mode of tension of the Ce-O, which corresponds to the active S1U mode in the IR for the fluorite structure, which is indicative of the presence of ceria. A large band observed at 3187 cm^−1^ is attributed to the vibration tension of the OH corresponding to residual water and hydroxyl groups. The bands at 1311 and 877 cm^−1^ is assigned to the vibrational stretching mode of H_2_O [48,52,64,65,66].

The FTIR spectra of the PU showed an absorbance peak at 3324 cm^−1^ due to N–H urethane stretching vibration. The peaks observed at 2954 and 2869 cm^−1^ are corresponding to asymmetric CH_2_ stretching and symmetric CH_2_ stretching respectively. A typical absorption band at 1714 cm^−1^ is assigned to C=O groups in the urethane unite. The peak at 1596 cm^−1^ could be attributed to the aromatic groups on the PU molecules [67].

The main characteristic bands of the plain PVA NFs that could be observed at 3200–3680, 2923, 1419, and 1085 cm^−1^, are assigned to (–OH stretching vibration), (C–H stretching), (CHOH), and (C–O stretching), respectively [23].

The spectroscopy of pure gelatin (Gel) showed characteristic peaks at 1626 (amide I) and 1525 cm^−1^ (amide II), which are corresponding to the stretching vibration of the C=O bond, and coupling of bending of N–H bond and stretching of C–N bonds, respectively. Peaks at 1442, 1333, and 1234 cm^−1^ are attributed to the C-H vibration, and C-N stretching, respectively [68,69,70,71].

It can be observed that pure-S1 showed the prominent and main characteristic peaks of PU, PVA, and Gel, with slight changes in intensities and band shifts. Thus, it confirmed that all characteristic peaks of the polymeric constituents (PU, PVA, and Gel) were recorded. As a result of the addition of CeNPs in nCeO_2_-S2, the broad beak at 3200–3750 cm^−1^ was reduced and shifted due to the hydrogen bonding between the surface of NPs and the –OH group. By embedding CEO into the PU (CEO/nCeO_2_S3 and CEO/nCeO_2_-S4), the characteristic absorption peaks of CEO were completely retarded, indicating that CEO was efficiently incorporated into the polymer solution. Furthermore, it can be noted that there were some changes in the intensities and band shifts due to the incorporation of the CEO. The peak between 3150 to 3700 cm^−1^ in both CEO/nCeO_2_-S3 and CEO/nCeO_2_-S4 was deep by the incorporation of CEO.

### 3.6. DSC Analysis

A DSC thermogram was performed (Figure 5B) to investigate the potential interactions of the polymeric electrospun layers (PU/PVA-Gel) and after incorporating nanoceria and CEO. The thermogram of the electrospun PU/PVA-Gel NFs (pure-S1) showed a curve from 30–81 °C, with a peak at 52.5 °C, which is attributed to Tg. The Tm of pure-S was recorded at 186 °C. After incorporating the nanoceria in nCeO_2_-S2, the Tg and Tm were observed at 47.5 and 186.5 °C, respectively. As a result of the addition of both CEO and nCeO_2_ in CEO/ nCeO_2_-S3, the thermal stability was improved where both Tg and Tm red-shifted to the higher temperature. Both Tg and Tm of CEO/nCeO_2_-S3 were recorded at 53.5 and 189.3 °C. The Tm and Tg of the thermogram of CEO/nCeO_2_-S4 were recorded at 54.5 and 194 °C, respectively. Additionally, there is a peak observed at 147 °C, which might be attributed to the interactions of the CEO with the polymers. It was reported that the addition of the CEO to the polymeric solution could improve its thermal stability [72]. Such results are consistent with swelling properties. It was reported that the addition of CEO to the PU electrospun layer and PVA/Gel-nCeO_2_ could produce a new compact sheet that is not easily detached and is more stable than incorporation of nanoceria only. Pure-S1 as drug-free NFs, nCeO_2_-S2 as nCeO_2_-loaded NFs, and CEO/nCeO_2_-S4 as CEO and nCeO_2_-loaded NFs were selected for further studies.

### 3.7. Antibacterial Activity

**Figure 6:**
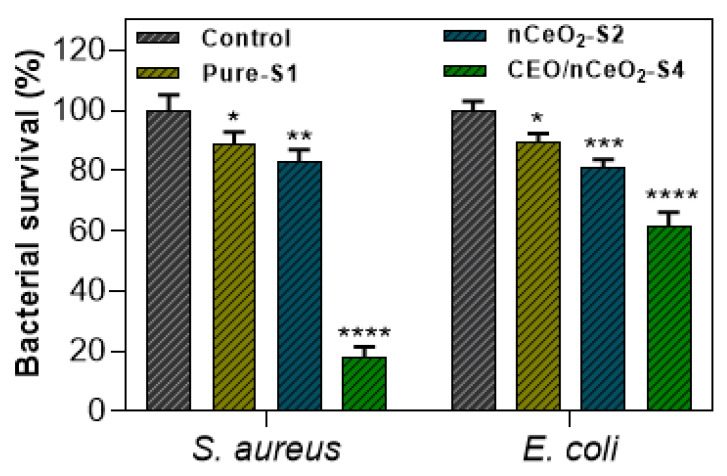
Antibacterial activity (Log CFU/mL) of Pure-S1, nCeO_2_-S1, CEO/nCeO_2_-S3, and CEO/nCeO_2_-S4, against *S. aureus* and *E. coli*. Data are expressed as average ± SD. * *p* < 0.0332; ** *p* < 0.0021, *** *p* < 0.0002, **** *p* < 0.0001, versus control.

As the designated NFs are utilized as wound dressing scaffolds, the antibacterial effect was an important assay. The wounded area is subjected to the contamination of Gram-positive and Gram-negative bacteria, which secrete toxic substances and cause septic wounds. Thus, the contaminated wound lesions do not heal properly, as the skin cells lyse and die in such an environment. Therefore, it is important to incorporate antibacterial agents into electrospun NFs to eradicate infectious bacteria and thus accelerate the wound healing process. Herein, the antibacterial activity of pure-S1, nCeO_2_-S2, and CEO/nCeO_2_-S4 were investigated against *S. aureus* as Gram-positive bacteria and *E. coli* as Gram-negative bacteria. The ratio of live bacteria against the total number of bacteria (both live and dead) was calculated to determine the antibacterial activity of the NF mats. As shown in Figure 6, the CEO/nCeO_2_-S4 showed lower bacterial viability against *S. aureus* (18.2%) than pure-S1 (88.7%) and nCeO_2_-S2 (82.6%). CEO/nCeO_2_-S4 showed weak antibacterial effectiveness against *E. coli* (with 61.8% survival rate), but was still more effective than pure-S1 and nCeO_2_-S2. The mild antibacterial activity of the produced NFs might be attributed to the slow release of the loaded agents. The results suggest that the antibacterial activity of NF mats was enhanced by the incorporation of CEO. It has been reported that oils, plant extracts, and their ingredients are more potent against Gram-positive bacteria than Gram-negative bacteria. Though Gram-positive bacteria are thicker (20–80 nm) than Gram-negative bacteria (10 nm), the complex structure of the cell wall of Gram-negative bacteria, in addition to the presence of the outer lipopolysaccharide capsule, render them more resistant. Moreover, CEO as hydrophobic can easily penetrate the cell walls of Gram-positive bacteria. Meanwhile, the external capsule of Gram-negative bacteria reduces the penetration of EOs into microbial cells [73]. Generally, the antibacterial activity of the EOs could be attributed to their capability to alter membrane permeability by destroying the electron transport system and eventually destroying microbial cells [74]. In another mechanism, EOs could downgrade protein functions through binding with the protein in the bacterial cell wall [75]. The mechanism of the antibacterial effect of CEO can be attributed to the presence of the active molecule cinnamaldehyde, which is electronegative charged and thus can interfere with nitrogen-containing substances, such as proteins and nucleic acids [76]. Cinnamaldehyde, followed by linalool, ortho-methoxycinnamaldehyde, β -caryophyllene, 1, 8-cineole, and eugenol were reported as the main constituents in the CEO with antibacterial activity [77]. The CEO can display other antibacterial mechanisms by inhibiting cell division, ATPase, and biofilm formation [78,79]. Zhang et al. investigated the antibacterial property of cinnamon EO and its mechanistic approach by understanding the mode of its antibacterial action. They stated that the CEO caused the leakage of small electrolytes. In addition, the concentration of proteins and nucleic acids in cell suspension rose with increased cinnamon EO. Furthermore, the bacterial metabolic activity decreased 3–5 fold [80]. According to Goni et al., cinnamaldehyde is the major constituent of the CEO [81]. Further studies are required to investigate the mechanism of the antibacterial activity of CEO and nanoceria.

In a previous study, PCL NFs loaded with 6% peppermint oil as wound dressings could decrease some bacterial viability after 24 h incubation [82]. In another study, PCL/gelatin NFs incorporated with 6% clove essential oil were applied as potential mats for wound healing applications where they displayed some inhibition for *S. aureus* and *E. coli* [83]. Cinnamaldehyde (the major component of the cinnamon essential oil, CEO) was applied topically at sub-inhibitory concentrations (MIC/2), which already showed a slight decrease in *Pseudomonas aeruginosa* but could decrease their metabolic rates and biofilm. Such sub-inhibitory concentrations were topically applied to wounded mice. In animals topically treated with cinnamaldehyde, healing was noted from day 3 post-wound induction, and the wound contracted by 62.3% at the 7th day post-lesion [84]. According to previous reports, the effective antibacterial materials should display inhibition at least 30% for the microbial concentration; thus, the prepared NFs could satisfy this feature, and could be considered efficient antibacterial agents [85,86].

### 3.8. Cytobiocompatability

**Figure 7:**
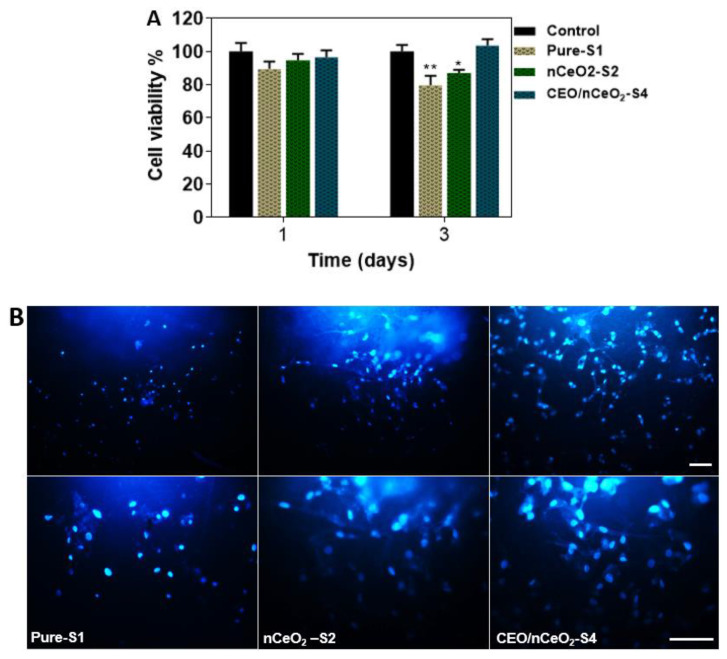
MTT assay of nanofibrous mats at 1 and 3 days, (**A**) and fluorescence images showing the proliferation of ADMSCs on the nanofibrous mats using DAPI staining after 3 days (**B**). Scale bar: 100 µm. Data are expressed as average ± SD. * *p* < 0.0332; ** *p* < 0.0021, versus control.

The MTT assay was performed to assess the biocompatibility of Pure-S1, nCeO_2_-S2, and CEO/nCeO_2_-S4 fiber mats after 1 and 3 days of incubation, as shown in Figure 7A. The results indicated that the cell viability of all fiber mats showed no significance after 1 day compared to the control. In addition, both nCeO_2_-S2 and CEO/nCeO_2_-S4 showed higher cell viability than pure-S1. After 3 days, there was a decrease in the cell viability for both pure-S1 and nCeO_2_-S2. On the other hand, the cell viability increased after 3 days for CEO/nCeO_2_-S2. The results revealed the biocompatibility of the platforms made of PU/PVA-Gel electrospun NFs with higher cell viability by the addition of CEO and nCeO_2_.

MSCs were used because they could be suitable for cell-laded scaffolds applied for tissue regeneration [22]. Employing MSCs for the treatment of wounds has been demonstrated based on their therapeutic benefits for tissue regeneration through secreting various factors, such as vascular endothelial growth factor (VEGF), EGF, fibroblast growth factor (FGF), keratinocyte growth factor (KGF), insulin-like growth factor (IGF), platelet-derived growth factor (PDGF), transforming growth factor beta (TGF-β), and hepatocyte growth factor (HGF) [87]. Many studies have tried to apply suitable platforms for maintaining the viability of MSC for wound healing.

The increase in cell viability upon the addition of nanoceria can be attributed to its antioxidant effect, which supports the cells to grow in an environment free of oxidative stress, which suppresses the growth of cells and damages them. Moreover, the addition of CEO along with nCeO_2_ as in nCeO_2_/CEO-S4 (PU-CEO/PVA-Gel-nCeO_2_), could demonstrate potent antioxidant activity. Thus, the MSCs could grow and increase more efficiently.

The growth rate of ADMSCs on the produced NFs was investigated by DAPI staining and inverted optical microscopy. Figure 7B shows the cell growth and distribution after 3 days over pure-S1, nCeO_2_-S2, and CEO/nCeO_2_-S4 electrospun layer-by layer NFs. It is observed that both nCeO_2_-S2 and CEO/nCeO_2_-S4 showed efficient platforms for the growth of the MSC in comparison to pure-S1. Moreover, the cells seeded over CEO/nCeO_2_-S4 showed a better growth rate with better distribution, and their colonies were larger compared to the cells seeded over nCeO_2_-S2.

Cinnamon extract was used for tissue regeneration (bone) through the proliferation effect of osteoblasts at concentrations up to 5%. The osteogenic effect of cinnamon extract through the induction of osteogenic markers runx2, coll, OSN, and osteocalcin [88]. In a study performed by Kossyvaki et al., the CEO was loaded in keratin fibrous patches at different ratios for skin burn care. The study demonstrated that cell viability was increased by increasing the fiber extract up to 0.1 mg/mL at all studied CEO ratios compared to CEO-free keratin fibrous patches. By increasing the CEO-loaded keratin to 0.5 mg/mL, the cell viability decreased [89]. The materials applied in the study PU, Gel, and PVA as electrospun NFs were reported for their support for cell adhesion, growth, and proliferation, where they simulate the structure of a natural extracellular matrix (ECM) [39,90].

The study used 4.5% CEO to ensure that this amount would be biocompatible. With this amount, it could show higher antibacterial activity in comparison to pure NFs. In conclusion, the presence of CEO with nanoceria in the prepared PU/PVA-Gel NFs, presented antibacterial activity and induced the cell population. Through these results, the electrospun PU/PVA-Gel could be a promising dressing material for chronic and diabetic wounds through the simultaneous and sustained delivery of nCeO_2_ and CEO. This study presented an initial trial to apply CEO along with nCeO_2_ in PU/PVA-Gel, where different amounts of CEO are recommended to apply with nCeO_2_, to study their effect at different ratios and select the optimum one that achieves the desired antibacterial effect with excellent biocompatibility.

## 4. Conclusions

Herein, we have reported, for the first time, the preparation and characterization of PU/PVA-Gel NFs incorporated with nCeO_2_ and CEO via the dual spinneret electrospinning technique. nCeO_2_ as a cell promotor was incorporated into PVA-Gel, where CEO as an antibacterial agent was loaded into PU NFs. The effect of CEO on the morphology, conformation, and thermal stability of PU/PVA-Gel NFs was investigated, and the results indicated that the addition of CEO improved the morphology, thermal stability, and mechanical strength of the NFs. Physical characterization, including porosity, swelling, and degradation tests, showed that the NFs have good porosity and suitable fluid uptake capability with a slow degradation rate. The antibacterial activity and biocompatibility, including cell viability and distribution, were improved in the presence of CEO when compared to PU/PVA-Gel and nCeO_2_ loaded PU/PVA/Gel NFs. Thus, this study suggests that the developed PU/PVA-Gel nanofibrous scaffolds could be candidate materials for wound dressings by incorporating nCeO_2_ and CEO. The present work paves the way for a further future study, including mechanistic investigations to elucidate the exact contribution of CEO and nCeO_2_, and to build up, enhance, and develop novel efficient nanoplatforms for healing of diabetic wound from the nCeO_2_/CEO-containing NFs. 

## Figures and Tables

**Table 1:** Composition of the prepared CEO/nCeO_2_ incorporating NFs.

Sample No.	PU Layer Solution		PVA-Gel Layer Solution				Nanofibers Sample Code
	PU (wr%)	CEO (*v*/*v*%)	PVA (wt%)	Gel (wt%)	PVA/Gel	nCeO_2_ (*w*/*w*%)	
PU	12	-	-	-	-	-	PU
S1	12	-	12	10	7/3	-	Pure-S1
S2	12	-	12	10	7/3	0.5	CEO/nCeO_2_-S2
S3	12	2.25	12	10	7/3	0.5	CEO/nCeO_2_-S3
S4	12	4.5	12	10	7/3	0.5	CEO/nCeO_2_-S4

## Data Availability

Data are contained within the article.
